# Prognostic value of a three-scale grading system based on combining molecular imaging with ^68^Ga-DOTATATE and ^18^F-FDG PET/CT in patients with metastatic gastroenteropancreatic neuroendocrine neoplasias

**DOI:** 10.18632/oncotarget.27460

**Published:** 2020-02-11

**Authors:** Ioannis Karfis, Gwennaëlle Marin, Hugo Levillain, Stylianos Drisis, Raoul Muteganya, Gabriela Critchi, Loubna Taraji-Schiltz, Carlos Artigas Guix, Leila Shaza, Meriem Elbachiri, Laura Mans, Godelieve Machiels, Alain Hendlisz, Patrick Flamen

**Affiliations:** ^1^ Nuclear Medicine Department, Institut Jules Bordet-Université Libre de Bruxelles (ULB), Brussels, Belgium; ^2^ Radiology/Medical Imaging Department, Institut Jules Bordet-Université Libre de Bruxelles (ULB), Brussels, Belgium; ^3^ Digestive Oncology Department, Institut Jules Bordet-Université Libre de Bruxelles (ULB), Brussels, Belgium

**Keywords:** gastroenteropancreatic neuroendocrine neoplasias, molecular imaging, prognostic biomarkers, ^68^Ga-DOTATATE PET/CT, ^18^F-FDG PET/CT

## Abstract

We investigated on the added prognostic value of a three-scale combined molecular imaging with ^68^Ga-DOTATATE and ^18^F-FDG PET/CT, (compared to Ki-67 based histological grading), in gastroenteropancreatic neuroendocrine neoplasia patients. 85 patients with histologically proven metastatic gastroenteropancreatic neuroendocrine neoplasias, who underwent combined PET/CT imaging were retrospectively evaluated. Highest Ki-67 value available at time of ^18^F-FDG PET/CT was recorded. Patients were classified according to World Health Organization/European Neuroendocrine Tumor Society histological grades (G1, G2, G3) and into three distinct imaging categories (C1: all lesions are ^18^F-FDG negative/^68^Ga-DOTATATE positive, C2: patients with one or more ^18^F-FDG positive lesions, all of them ^68^Ga-DOTATATE positive, C3: patients with one or more ^18^F-FDG positive lesions, at least one of them ^68^Ga-DOTATATE negative). The primary endpoint of the study was Progression-Free Survival, assessed from the date of ^18^F-FDG PET/CT to the date of radiological progression according to Response Evaluation Criteria In Solid Tumors version 1.1. Classification according to histological grade did not show significant statistical difference in median Progression-Free Survival between G1 and G2 but was significant between G2 and G3 patients. In contrast, median Progression-Free Survival was significantly higher in C1 compared to C2 and in C2 compared to C3 patients, revealing three distinctive imaging categories, each with highly distinctive prognosis. Our three-scale combined ^68^Ga-DOTATATE/^18^F-FDG PET imaging classification holds high prognostic value in patients with metastatic gastroenteropancreatic neuroendocrine neoplasias.

## INTRODUCTION

GastroEnteroPancreatic NeuroEndocrine Neoplasias (GEP NENs) represent a challenging clinical entity with marked heterogeneity in terms of prognosis, behavior and evolution over time. Approximately half of the GEP NEN patients have already developed distant metastases at the time of primary staging [1] with liver being the predominant localization of tumor spread [2]. Surgical resection of the primary and the metastases, when feasible, remains the only curative treatment in patients with GEP NENs. Accurate assessment of the disease extent and the prognosis are crucial in order to optimize disease management.

The choice between available therapeutic modalities mainly relies on the histological assessment of tissue samples. Anatomo-pathological reports provide information about tumor size, extent of local invasion, presence of nodal metastases, but also differentiation status and tumor grade [3]. The tumor grade relies on both, the percentage of neoplastic cells expressing the Ki-67 protein (during the active phases of the cell cycle) and the mitotic count and has been shown to provide significant prognostic information [1, 4]. Current grading classifications endorsed by World Health Organization/European Neuroendocrine Tumor Society (2010 for all GEP NENs [5] and 2017 for pancreatic NENs [6]) rely on the use of Ki-67 labeling index and/or the mitotic rate. NENs showing a well-differentiated histology and a low proliferation rate have been called Neuroendocrine Tumors (NETs). They often present with an indolent behavior and most express somatostatin receptors (SSTR) on their surface [7]. In contrast, NENs displaying a poorly differentiated histology and a high proliferation rate are called Neuroendocrine Carcinomas (NECs). Their natural history is aggressive and they fail to express SSTR. Several issues regarding Ki-67 assessment have been raised, concerning mainly sampling errors due to tumoral heterogeneity and inter-observer variability, which may provide conflicting information about grading [8, 9].

Positron Emission Tomography (PET) imaging, by means of ^68^Ga-DOTA-agonists and ^18^F-FDG, represent a powerful nuclear medicine tool for management of patients with GEP NENs, targeting differentiation status (via overexpression of trans-membrane SSTR, specific to the neuroendocrine phenotype) and tumoral aggressiveness (via glycolytic metabolism, a nonspecific energetic pathway of neuroendocrine neoplasias). Combined imaging by those two tracers, may highlight the intimate relationship between SSTR expression and metabolism, allowing an *in vivo* whole-body phenotypic characterization of the disease.

The primary objective of this study was to develop an easy-to-implement imaging score for combined molecular imaging (^68^Ga-DOTATATE/^18^F-FDG PET) reflecting tumoral heterogeneity in patients with metastatic GEP NENs and to assess its potential prognostic value. The secondary objective was to compare its prognostic value with that of the Ki-67 based histological grade (as available at the date of PET imaging).

## RESULTS

**Figure 1:**
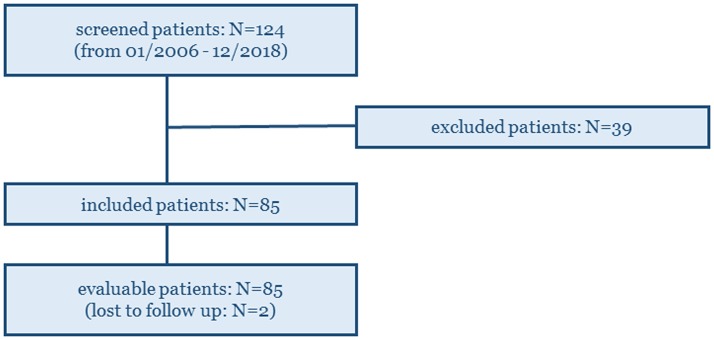
124 GEP NENs patients who underwent combined ^68^Ga-DOTATATE and ^18^F-FDG PET/CT imaging in our Institute (within a maximum time window of three months between them), were screened prior to inclusion. 85 patients were finally included and evaluated on the study.

124 in total GEP NENs patients who underwent combined ^68^Ga-DOTATATE and ^18^F-FDG PET/CT imaging, between January 2008 and December 2018 within a maximum time window of three months between them, were screened prior to inclusion. 39 patients were excluded (no morphologically measurable target lesions: 12 patients, unknown primary NENs: 3 patients, surgery with curative intent after the two PETs: 19 patients, and second primary malignancies: 5 patients) and 85 patients were finally included and evaluated (Figure 1), including 2 patients that were lost to follow up (for which however, survival data were available).

The cohort characteristics are summarized in Table 1. The median time between the date of the highest available Ki-67 index and the date of the ^18^F-FDG PET was 22.7 months [range: 0.1–126.4 months]. The median age of the patients was 63 years [range: 17–81 years]. The median follow-up of the entire cohort was 20.8 months [range: 0–99.6 months, first quartile: 7.1-third quartile: 38.1 months].

**Table 1:** Cohort summary characteristics

Characteristic	Number of patients (%)
**Site of Primary Tumor**	
large bowel	01 (1.2%)
pancreas	29 (34.1%)
rectum	07 (8.2%)
small bowel	46 (54.1%)
stomach	02 (2.4%)
**Gender**
female	47 (55%)
male	38 (45%)
**Histological Classification**	
G1	21 (24.7%)
G2	46 (54.1%)
G3	18 (21.2%)
**Combined PET Classification**	
C1	28 (32.9%)
C2	46 (54.1%)
C3	11 (13%)

Legend: Summary of the cohort characteristics (site of primary tumor, gender, histological and combined PET classification).

According to the histological classification, 21 patients were graded as G1, 46 as G2 and 18 as G3. According to the combined imaging classification, 28 patients were C1, 46 were C2 and 11 patients were C3 (Table 2). The agreement between the two classification systems was poor with a Kendall’s Rank Correlation Coefficient *τ* of -0.003 [38/85 (44.7%)].

**Table 2:** Agreement table between the two classification systems

	G1	G2	G3	Total
**C1**	9	18	1	28
**C2**	12	23	11	46
**C3**	0	5	6	11
**TOTAL**	21	46	18	85

Legend: The agreement between the two classification systems was poor

**Figure 2:**
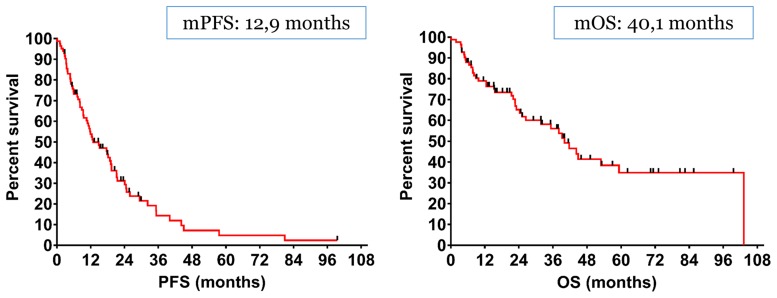
The median Progression-Free Survival (mPFS) and the median Overall Survival (mOS) of the entire cohort was 12.9 months and 40.1 months respectively.

The median Progression-Free Survival (mPFS) and the median Overall Survival (mOS) of the entire cohort was 12.9 months and 40.1 months respectively (Figure 2).

**Figure 3:**
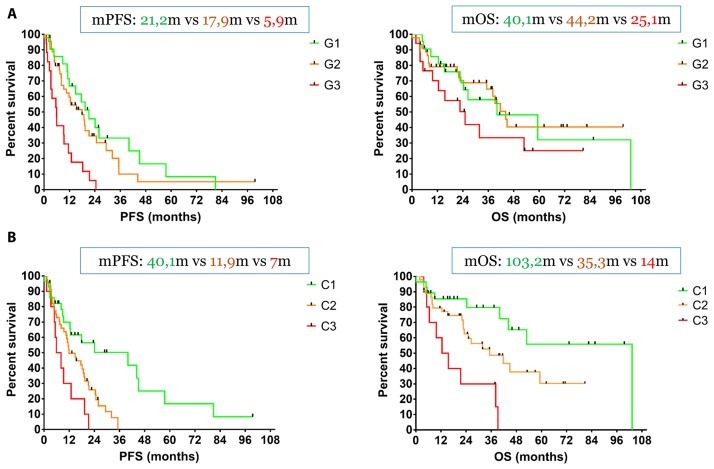
(**A**) The mPFS values of G1, G2 and G3 patients were 21.2 months, 17.9 months and 5.9 months respectively. We did not observe any statistical difference between G1 and G2 patients, while G2 patients had a statistically significant longer PFS compared to G3. Accordingly, the mOS values of G1, G2 and G3 patients were 40.1 months, 44.2 months and 25.1 months respectively and no statistical difference between any of the three categories was observed. (**B**) The three survival curves were completely separated with statistically different mPFS values of 40.1 months (C1 patients), 11.9 months (C2 patients) and 7.0 months (C3 patients) respectively. Similarly, C1 patients had a mOS of 103.2 months, while C2 and C3 patients demonstrated a mOS of 35.3 months and 14.0 months respectively. There was a trend toward significance between C1 and C2 patients and C3 patients had a significantly lower mOS compared to C2.

Regarding histological grade classification, the mPFS values of G1, G2 and G3 patients were 21.2 months, 17.9 months and 5.9 months respectively. We did not observe any statistical difference between G1 and G2 patients [Hazard for G1/G2: *p* = 0.34, Hazard Ratio HR: 0.75 (95%CI, 0.42–1.34)], while G2 patients had a statistically significant longer PFS compared to G3 [Hazard for G2/G3: *p* < 0.001, HR: 0.37 (95%CI, 0.17–0.79)], just as G1 compared to G3 [Hazard for G1/G3: *p* < 0.001, HR: 0.33 (95%CI, 0.15–0.72)]. Accordingly, the mOS values of G1, G2 and G3 patients were 40.1 months, 44.2 months and 25.1 months respectively, while no statistical difference between any of the three categories was observed [Hazard for G1/G2: *p* = 0.93, HR: 1.03 (95%CI, 0.49–2.20)], [Hazard for G2/G3: *p* = 0.10, HR: 0.55 (95%CI, 0.24–1.23)] and [Hazard for G1/G3: p = 0.12, HR: 0.54 (95%CI, 0.23–1.26)] (Figure 3A).

Regarding combined imaging classification, the three survival curves were completely separated with statistically different mPFS values of 40.1 months (C1 patients), 11.9 months (C2 patients) and 7.0 months (C3 patients) respectively: [Hazard for C1/C2: *p* = 0.004, HR: 0.47 (95%CI, 0.27–0.79)], [Hazard for C2/C3: *p* = 0.036, HR: 0.49 (95%CI, 0.20–1.19)] and [Hazard for C1/C3: p = 0.002, HR: 0.32 (95%CI, 0.11–0.90)]. Similarly, C1 patients had a mOS of 103.2 months, while C2 and C3 patients demonstrated a mOS of 35.3 months and 14.0 months respectively. There was a trend toward significance between C1 and C2 patients [Hazard for C1/C2: *p* = 0.08, HR: 0.51 (95%CI, 0.25–1.04)], while C3 patients had a significantly lower mOS compared to C2 [Hazard for C2/C3: *p* = 0.013, HR: 0.39 (95%CI, 0.14–1.09)] and obviously C1 [Hazard for C1/C3: *p* < 0.001, HR: 0.21 (95%CI, 0.06–0.70)] (Figure 3B).

## DISCUSSION

One of the landmarks of Neuroendocrine Differentiation is the overexpression of SSTR on the cellular surface, a particularly attractive target for imaging and therapy (the “theragnostic” approach) [10]. Imaging with radiolabeled somatostatin analogs (agonists), should be PET-based (e. g. ^68^Ga-DOTA-peptides), taking into account the numerous advantages over the classical somatostatin receptor scintigraphy [11]. Accurate staging of NENs is crucial, and the reported pooled sensitivity and specificity of ^68^Ga-DOTA PET imaging is 96% and 100%, respectively [11, 12]. It has been clearly documented that the expression of SSTR correlates strongly to the degree of differentiation of NENs [13] and the intensity of tracer uptake in ^68^Ga-DOTA PET correlates with SSTR expression [14]. Therefore, uptake is higher in well-differentiated NETs compared to poorly-differentiated NECs. In addition, the uptake in ^68^Ga-DOTA PET measured by maximun Standardized Uptake Value (SUVmax), holds a high prognostic value, as values of 19.3 or more, correlate with improved OS compared to those with poorer uptake [14]. Sharma et al [15], reported a lower cut-off in ^68^Ga-DOTA PET uptake, still with similar prognostic significance. Finally and as a consequence, SSTR imaging can select patients that qualify for treatment with somatostatin analogues and Peptide Receptor Radionuclide Therapy (PRRT).

Traditionally, ^18^F-FDG PET has been used for staging purposes in poorly-differentiated NECs or in case of negative SSTR imaging [16, 17], as poorly-differentiated NENs lose their ability to express SSTR. However, several prospective data underlined its strong independent prognostic value in NENs, even in those with low histological grade. In the pioneering study of Garin et al [18, 19], 36 patients with documented well-differentiated metastatic GEP NENs were offered a period of watch-and-wait before treatment initiation. Fifteen patients had a positive ^18^F-FDG PET and their median OS was only 15 months versus 119.5 months for patients with negative ^18^F-FDG PET. This difference was still significant for patients with positive SRS, highlighting a huge impact on survival of ^18^F-FDG uptake. Similarly, Binderup et al [20] investigated prospectively on 98 patients with metastatic NENs, of which, 92 of GEP origin. In this study, the prognostic value of several biomarkers (presence of hepatic metastases, Chromogranin A, Ki-67 based proliferation index and ^18^F-FDG uptake quantified by SUVmax) was assessed. During a follow-up of 12 months, 13 of the 14 deceased patients were ^18^F-FDG PET positive and a positive ^18^F-FDG PET was associated with a significantly higher risk of death with a HR of 10.3. The prognostic value of ^18^F-FDG PET outperformed the prognostic value of the rest of the evaluated biomarkers, results which were confirmed prospectively by Sansovini et al [21] and Nilica et al [22]. Finally, Johnbeck et al [23] assessed prospectively the prognostic value of ^18^F-FDG PET in 100 NENs patients (77 with GEP NENs). Patients with a positive ^18^F-FDG PET had a significantly worse prognosis (in terms of PFS and OS) than patients with a negative ^18^F-FDG PET.

Ezziddin et al [24] reviewed retrospectively data from 89 patients with metastatic GEP NENs and identified three distinctive prognostic groups based on the ratio of SUVmax of the lesion with the highest ^18^F-FDG uptake to the SUVmean of the normal hepatic parenchyma (ratio≤1; 1<ratio<2.3; ratio≥2.3). These groups were associated with significant differences in OS (mOS not reached after 114 months of follow up versus 55 months versus 13 months). That being said, ^18^F-FDG PET must be considered not only as a staging, but also (and perhaps most importantly), as a grading whole-body imaging tool, in which ^18^F-FDG PET positive lesions had a significant impact on prognosis, regardless of the expression of SSTR.

One issue that should be validated in larger studies is the definition of ^18^F-FDG positivity. In our cohort, we define as ^18^F-FDG positive any lesion with tracer uptake superior to the uptake of local background. In the study of Garin et al [18, 19], an SUVmax cutoff of 4.5 was used to define ^18^F-FDG positivity, whereas Sansovini et al [21] applied an arbitrary SUVmax cutoff of 2.5 separating ^18^F-FDG positive from ^18^F-FDG negative lesions. Whether low ^18^F-FDG uptake lesions represent an evolution step toward overt high uptake lesions or they just reflect a different pathophysiological underlying process (such as hypoxia [25]), remains to be investigated.

Combined molecular imaging with ^68^Ga-DOTA PET and ^18^F-FDG PET assesses simultaneously the tumor biology (SSTR expression and glycolytic metabolism). Different lesions can exhibit different degrees of tracer uptake and hence different degrees of differentiation and aggressiveness. These patterns of tumor heterogeneity reflect the precise phenotype of GEP NENs at any given moment in the course of the disease. Although both PETs provide complementary information, the major decision-making examination is ^18^F-FDG PET, (alias the metabolic fingerprint of the disease) and chances to encounter ^18^F-FDG-avid lesions or a mismatch in favor of ^18^F-FDG, increase with tumor grade.

We developed an easy-to-implement three-scale grading score for combined molecular imaging (^68^Ga-DOTATATE and ^18^F-FDG PET) based on the spatial distribution of the lesions and the relative uptake of the respective tracers. We classified our cohort into three distinctive imaging categories, each with highly distinctive prognosis. This was in contrast to the classification based on the histological grade, obviously due to the size of our cohort, since high volume epidemiological studies have validated the actual histological grades. Ki-67 based histological grade plays a prominent role in NENs management as it is worldwide, the most common tissue-based prognostic biomarker and has become essential for guiding therapy [26, 27]. However, if the biopsies leading to Ki-67 indexes determination are not PET-guided, they might be subject to sampling error, leading thus to underscores which are not reflecting necessarily the real tumor aggressiveness [28]. Thirteen patients in our cohort (15%), histologically labelled as G1, had ^18^F-FDG-avid lesions, but this percentage in low grade NETs can be as high as 40% [20, 22]. Moreover, Ki-67 index may evolve in the same patient over time and site [29, 30], affecting not only treatment decision but also disease prognosis [30]. In some cases, Ki-67 indexes in our cohort were as old as 10 years. If dictated by clinical scenarios, combined PET imaging should be repeated over time, in order to detect and reveal aggressive ^18^F-FDG-avid lesions. An accurate selection of (re-) biopsy sites can be the link between PET-driven biopsies and biopsy-driven treatments, optimizing NENs management and helping selection between different therapeutic options and strategies.

Chan et al [31], on their seminal study, classified 62 patients who performed ^68^Ga-DOTATATE and ^18^F-FDG PET within 31 days of each other, into five imaging categories (P1-P5), introducing the NETPET score. P1 indicated purely ^68^DOTATATE-avid disease without ^18^F-FDG uptake in any lesion (corresponding to our C1 category), while P5 indicated the exact opposite, pure ^18^F-FDG-avid and ^68^Ga-DOTATATE-negative lesions (corresponding to our C3 category). Particular attention must be paid to the intermediate categories (P2-P4), where lesions are avid into both tracers. Schematically, P2 comprises a pattern of ^18^F-FDG<^68^Ga-DOTATATE (mismatch in favor of ^68^Ga-DOTATATE), P3 a pattern of ^18^F-FDG = ^68^Ga-DOTATATE (no mismatch, all lesions are ^18^F-FDG and ^68^Ga-DOTATATE avid) and P4 a pattern of ^18^F-FDG>^68^Ga-DOTATATE (mismatch in favor of ^18^F-FDG). Our classification was slightly different: C2 comprised P2 and P3 patients, while in C3 we include P4 (minor/major mismatches in favour of ^18^F-FDG) along with P5 patients (complete mismatch in favour of ^18^F-FDG). We demonstrated that any mismatch in favour of ^18^F-FDG can significantly impact patient survival.

These two imaging classification systems (the NETPET and the one presented in our article) combine the biological information provided by the two PETs into a single classification scheme with high prognostic significance, with the ultimate purpose to identify mismatches in favor of ^18^F-FDG, as they represent the most aggressive phenotype of the disease. Nevertheless, the three-scale classification is easier to implement, was tested into a larger cohort of patients and provided a complete survival information, not only in terms of PFS but also in terms of OS.

Besides their prognostic significance, these systems may have further implications on therapeutic management. C1 patients have a low-grade and indolent disease. In contrast, C3 patients have high-grade, metabolically active disease and therefore warrant aggressive treatment. PRRT is suitable for C1 patients and in C2 patients as well. In C3 patients, the ^18^F-FDG positive/^68^Ga-DOTATATE negative part of the disease will be less affected by beta-irradiation of the therapeutic radio-isotopes (for instance by cross-irradiation from neighbouring ^68^Ga-DOTATATE positive lesions, if any), or not at all. PRRT therefore is not suitable for that patient category. Emerging data suggest the use of PRRT in combination with chemotherapeutic agents (such as temozolomide/capecitabine) in spatially concordant ^18^F-FDG positive disease [32, 33], in an attempt to boost the therapeutic efficacy of the former. Hence, Peptide Receptor Chemo-Radionuclide Therapy (PRCRT), can achieve unexpectedly long PFS, modifying the poor prognosis associated with ^18^F-FDG-avidity [34].

In summary, the combined ^68^Ga-DOTATATE/ ^18^F-FDG PET imaging classification presented on this article represent the precise phenotype of GEP NENs at any given moment of the disease and holds high prognostic value, compared to classification based on the histological grade. Its value as prognostic imaging biomarker should be further confirmed within prospective trials and tested multi-centrically to establish inter-rater reliability.

## MATERIALS AND METHODS

### Patient cohort and study design

Patients with histologically confirmed metastatic GEP NENs who underwent in our center combined imaging with ^68^Ga-DOTATATE and ^18^F-FDG PET/CT within a maximum window of 3 months were retrospectively included on the study. Patients with no morphologically measurable target lesions according to Response Evaluation Criteria In Solid Tumors (RECIST version 1.1) [35], patients with unknown primary NENs, patients who underwent surgery with curative intent after the two PETs and patients with second primary malignancies (or history of other active malignant disease) unless in remission for at least 5 years were excluded. Ki-67 index available at the day of ^18^F-FDG PET/CT was considered for patient categorization. The highest value of Ki-67 was considered if several values were available. The oldest pair of PETs was considered if more than one imaging pair was available. The study was approved by the local Ethics Committee of Jules Bordet Institute (CE2531).

The primary endpoint of the study was Progression-Free Survival (PFS), defined as from the date of ^18^F-FDG PET/CT, to the date of the documentation of morphological disease progression according to RECIST 1.1 on a Computerized Tomography (CT) or on a Magnetic Resonance Imaging (MRI). Baseline CT or MRI had to be performed no longer than 8 weeks prior or after ^18^F-FDG PET/CT. In case of non-availability of a baseline CT or MRI within the required time period, the CT of the ^18^F-FDG PET/CT was used, provided the presence of morphologically measurable lesions according to RECIST 1.1. Similarly, the CT of a follow up PET/CT was used for assessment of disease progression.

The secondary endpoint of the study was Overall Survival (OS), defined as from the date of ^18^F-FDG PET/CT to the date of death or the date of the last follow-up.

### Imaging

All ^18^F-FDG and ^68^Ga-DOTATATE PET/CT images were acquired at the Nuclear Medicine Department of Jules Bordet Institute using a General Electric (GE-Healthcare) Discovery 690 Time of Flight (TOF) PET system. Before ^18^F-FDG injection, patients had to fast for more than 6 hours and blood glucose level had to be lower than 150 mg/dL. Long acting somatostatin analogs were discontinued at least 4 weeks prior to ^68^Ga-DOTATATE PET/CT acquisition.

Whole-body PET images were acquired 60 min after injection [range: 51–88 min] of 3.81 MBq/Kg [range: 1.91–5.56 MBq/Kg] of ^18^F-FDG with 8 bed positions of 90 seconds with an overlap of 23.4%, and 61 min [range: 59–106 min] after injection of 2.00 MBq/Kg [range: 0.52–3.85 MBq/Kg] of ^68^Ga-DOTATATE with 10 bed positions of 150–180 seconds with an overlap of 23.4%. PET images were reconstructed with GE build-in algorithms; VUE Point FX for ^18^F-FDG [Ordered Subset Expectation Maximization (OSEM) algorithm with 2 iterations and 18 subsets, 6.4 mm Full-Width at Half-Maximum (FWHM) Gaussian post-filtering, and TOF attenuation and scatter corrections] and VUE Point FXS for ^68^Ga-DOTATATE (OSEM algorithm with 3 iterations and 18 subsets, 6.8 mm FWHM Gaussian post-filtering, and TOF, attenuation, scatter and resolution recovery corrections).

CT was performed with 64 slices helical scanner (VCT; GE Medical Systems). The tension was 120 kV, and the current was modulated by the Auto-mA software with a noise index of 30 (range: 30–200 mA) and the Adaptive Statistical Iterative Reconstruction (ASIR) algorithm. The other CT acquisition parameters were 0.5 s per CT rotation and a pitch of 0.98. The CT images were reconstructed with ASIR algorithm set at 40%, with a matrix of 512c×c512 (0.97c×c0.97 mm pixel size) and a slice thickness of 2.5cmm. The PET matrix was 192c×c192 pixels of 2.73c×c2.73 mm with a slice thickness of 3.27 mm.

### Patient classification systems

**Figure 4:**
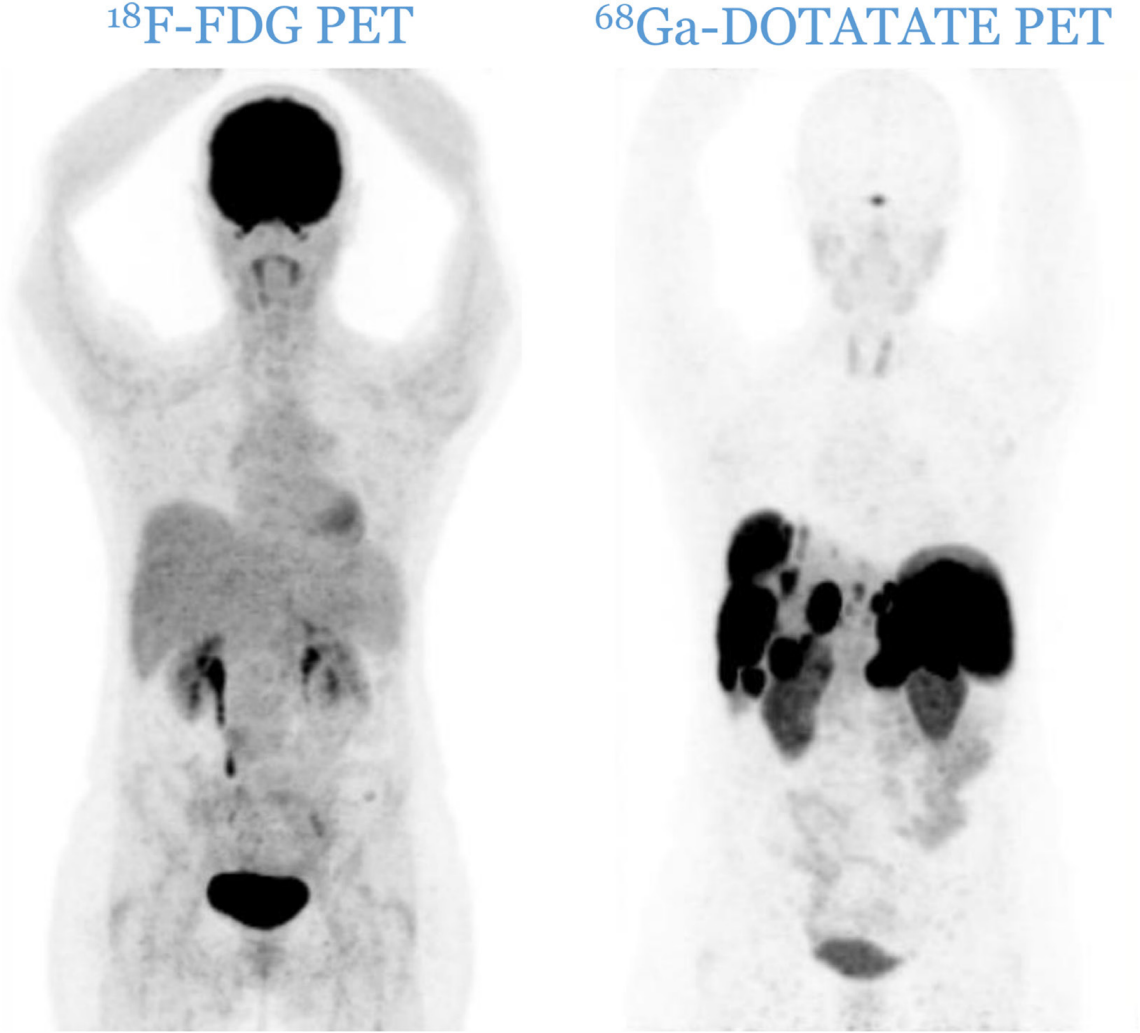
C1 category: all lesions are ^18^F-FDG negative and ^68^Ga-DOTATATE positive.

**Figure 5:**
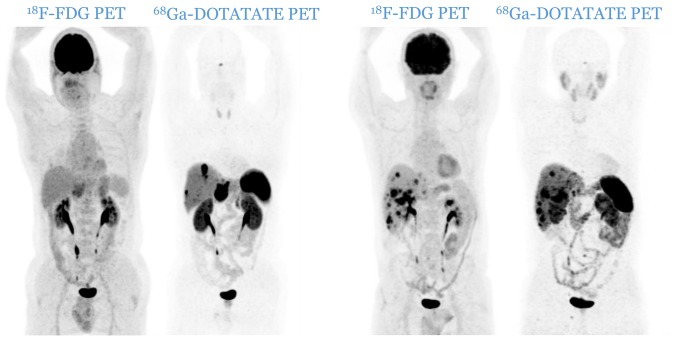
Two cases of patients from C2 category (two pairs of PETs): patients with one or more ^18^F-FDG positive lesions, all of them ^68^Ga-DOTATATE positive. There is no mismatch in favor of ^18^F-FDG.

**Figure 6:**
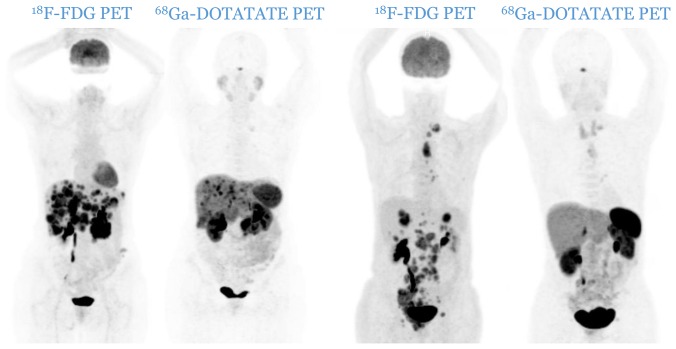
Two cases of patients from C3 category (two pairs of PETs): patients with one or more ^18^F-FDG positive lesions, all of them ^68^Ga-DOTATATE negative. A mismatch in favor of ^18^F-FDG is observable.

Patients were divided according to histological grade [5, 6] into three distinctive categories: G1, G2 and G3. The imaging classification was based upon the spatial distribution of the lesions and the relative uptake of the respective tracers. Anonymized PET image-sets were automatically co-registered anatomically, displayed simultaneously in transverse, sagittal and coronal planes and initially windowed with preset values for Standardized Uptake Value (SUV) of 0–15 for ^68^Ga-DOTATATE PET and SUV of 0–7 for ^18^F-FDG PET (AW Server 3.2, GE Healthcare). In both PETs, a lesion was considered as positive if tumoral uptake was superior to the local background. Patients were therefore divided into three distinct imaging categories: C1 (all lesions are ^18^F-FDG negative and ^68^Ga-DOTATATE positive, Figure 4), C2 (patients with one or more ^18^F-FDG positive lesions, all of them ^68^Ga-DOTATATE positive, Figure 5) and C3 (patients with one or more ^18^F-FDG positive lesions, at least one of them ^68^Ga-DOTATATE negative, Figure 6). Each pair of PETs was classified by two experienced nuclear medicine physicians into one of the three aforementioned categories (reporting was performed simultaneously). Radiological progression according to RECIST 1.1 was assessed without knowledge of the respective histological or imaging classification.

### Statistical analysis

Assessments were performed using GraphPad Prism 7 (GraphPad Software Inc., California USA), with a statistical significance level at *p* ≤ 0.05. Kaplan-Meier survival curves were constructed and compared using the log-rank test. The agreement between histological grade and combined PET imaging was assessed using the Kendall’s Rank Correlation Coefficient.

## Abbreviations

ASIR: Adaptive Statistical Iterative Reconstruction
CT: Computerized Tomography
FWHM: Full-Width at Half-Maximum
GEP NENs: GastroEnteroPancreatic NeuroEndocrine Neoplasias
HR: Hazard Ratio
MRI: Magnetic Resonance Imaging
NECs: Neuroendocrine Carcinomas
NETs: Neuroendocrine Tumors
OS: Overall Survival
OSEM: Ordered Subset Expectation Maximization
PET: Positron Emission Tomography
PFS: Progression-Free Survival
PRCRT: Peptide Receptor Chemo-Radionuclide Therapy
PRRT: Peptide Receptor Radionuclide Therapy
RECIST: Response Evaluation Criteria In Solid Tumors
SRS: Somatostatin Receptor Scintigraphy
SSTR: Somatostatin Receptors
SUV: Standardized Uptake Value
TOF: Time of Flight
